# 5-Aminolevulinic Acid (ALA) Alleviated Salinity Stress in Cucumber Seedlings by Enhancing Chlorophyll Synthesis Pathway

**DOI:** 10.3389/fpls.2018.00635

**Published:** 2018-05-15

**Authors:** Yue Wu, Xin Jin, Weibiao Liao, Linli Hu, Mohammed M. Dawuda, Xingjie Zhao, Zhongqi Tang, Tingyu Gong, Jihua Yu

**Affiliations:** ^1^College of Horticulture, Gansu Agricultural University, Lanzhou, China; ^2^Department of Horticulture, Faculty of Agriculture, University for Development Studies, Tamale, Ghana; ^3^School of Environmental and Municipal Engineering, Lanzhou Jiaotong University, Lanzhou, China

**Keywords:** 5-aminolevulinic acid, salinity, photosynthesis, tetrapyrrol biosynthesis, cucumber seedlings

## Abstract

5-Aminolevulinic acid (ALA) is a common precursor of tetrapyrroles as well as a crucial growth regulator in higher plants. ALA has been proven to be effective in improving photosynthesis and alleviating the adverse effects of various abiotic stresses in higher plants. However, little is known about the mechanism of ALA in ameliorating the photosynthesis of plant under abiotic stress. In this paper, we studied the effects of exogenous ALA on salinity-induced damages of photosynthesis in cucumber (*Cucumis sativus* L.) seedlings. We found that the morphology (plant height, leave area), light utilization capacity of PS II [qL, Y(II)] and gas exchange capacity (Pn, gs, Ci, and Tr) were significantly retarded under NaCl stress, but these parameters were all recovered by the foliar application of 25 mg L^-1^ ALA. Besides, salinity caused heme accumulation and up-regulation of gene expression of ferrochelatase (*HEMH*) with suppression of other genes involved in chlorophyll synthesis pathway. Exogenously application of ALA under salinity down-regulated the heme content and *HEMH* expression, but increased the gene expression levels of glutamyl-tRNA reductase (*HEMA1*), Mg-chelatase (*CHLH*), and protochlorophyllide oxidoreductase (*POR*). Moreover, the contents of intermediates involved in chlorophyll branch were increased by ALA, including protoporphyrin IX (Proto IX), Mg-protoporphyrin IX (Mg-Proto IX, protochlorophyllide (Pchlide), and chlorophyll (Chl *a* and Chl *b*) under salt stress. Ultrastructural observation of mesophyll cell showed that the damages of photosynthetic apparatus under salinity were fixed by ALA. Collectively, the chlorophyll biosynthesis pathway was enhanced by exogenous ALA to improve the tolerance of cucumber under salinity.

## Introduction

5-aminolevulinic acid (ALA) has been considered as a growth regulator or potential plant hormone in higher plants. Its promotive role in enhancing plant biomass, photosynthesis and fruit quality under normal growth condition has been confirmed in rice (*Oryza sativa* L.) (Nguyen et al., 2016), strawberry (*Fragaria ananassa* Duch.) (Sun et al., 2017), and peach (*Prunus persica* L.)(Ye et al., 2017). In addition, ALA is known to be effective against the harmful effects caused by various abiotic stresses in plants. For example, foliar application of ALA alleviated the peroxidation of membrane and inhibition of net photosynthetic rate caused by salinity stress in creeping bentgrass (*Agrostis stolonifera* L.) (Yang et al., 2014). The application of ALA to roots significantly reduced the harmful effects of waterlogging stress by enhancing the activities of lactate dehydrogenase (LDH) and alcohol dehydrogenase (ADH) in *Ficus carica* L. (An et al., 2016). Moreover, exogenous ALA improved the resistance of peach (*Prunnus persica* L.) (Ye et al., 2016), tomato (*Lycopersicon esculentum* Mill.) (Zhang Z.-P. et al., 2015), rice (*Oryza sativa* L.) (Nunkaew et al., 2014), swiss chard (*Beta vulgaris* L.) (Liu et al., 2014), sicklepod (*Cassia obtusifolia* L.) (Zhang et al., 2013), and cucumber (*Cucumis sativus* L.) (Zhen et al., 2012) to salt stress. Furthermore, as a key precursor in the biosynthesis pathway of chlorophyll, ALA was found to have promotive role in photosynthesis under various stresses. Exogenously supplied ALA increased the content of chlorophyll which was suppressed by UV-B stress in lettuce (*Lactuca sativa* L.) (Aksakal et al., 2017). In another study, foliar application of ALA up-regulated the chlorophyll fluorescence indexes, including qP, φPSII, and Fv/Fm, in oilseed rape (*Brassica napus* L.) under drought stress (Liu et al., 2013). Besides, gas exchange indexes, such as net photosynthetic rate (Pn), stomatal conductance (gs), intercellular CO_2_ concentration (Ci) and transpiration rate (Tr), which were adversely affected by abiotic stress, were, however, promoted by ALA application in cauliflower (*Brassica oleracea botrytis* L.) under chromium stress (Ahmad et al., 2017). The relative gene expressions, like fructose-1,6-bisphosphatase (*FBP*), triose-3-phosphate isomerase (*TPI*) and ribulose-1,5-bisphosphate carboxylase/oxygenase small subunit (*RBCS*) of enzymes in Calvin cycle of photosynthesis were up-regulated by ALA and the carbohydrate contents were enhanced in oilseed rape (*Brassica napus* L.) under drought stress (Liu et al., 2016a).

Recently, exogenous application of ALA has been shown to have a positive effect on chlorophyll synthesis in de-etiolated cotyledon of oilseed rape under water-deficit stress (Liu et al., 2016b). Moreover, as another metabolic branch downstream of ALA, endogenous heme content was increased significantly by exogenous ALA in maize (*Zea mays* L.) under non-stressful conditions (Yonezawa et al., 2015). However, the regulative mechanisms of exogenous ALA to tetrapyrrol biosynthesis pathway and photosynthesis under salt stress have not been evaluated yet. Keeping in view of the crucial role ALA playing in tetrapyrrol synthesis and its alleviative effects to stress-damages in plant, the present study was designed to test a hypothesis that exogenous ALA could enhance plant stress tolerance by heightening the chlorophyll synthesis pathway. In this paper, the intermediate contents and relative gene expression levels of crucial enzymes among branches downstream of ALA metabolic pathway (including Fe-branch and Mg-branch) in cucumber under salinity stress were determined. Then, the photosynthesis capacity, intrinsic water use efficiency and the ultrastructure in mesophyll cell of cucumber leaves were determined to verify the stimulative effects of ALA. Thus, the main objective of the study was to explore the mechanism of ALA in improving plants tolerance to salt stress using cucumber as a test crop.

## Materials and Methods

### Plant Material and Growth Conditions

Cucumber seeds (*Cucumis sativus* L. cv. Xinchun No. 4) were surface sterilized with liquor potassii permanganatis (0.03%) for 10 min, and rinsed with distilled water. The seeds were soaked in distilled water for 6 h and then exposed to germination conditions. The moistened seeds were placed on double-layer filter paper and kept at 28 ± 1°C under dark condition. At 5 days after germination, seedlings with uniform size, fully spread cotyledons, and well-formed roots were transferred to 1-L opaque plastic containers containing half-strength Yamasaki’s cucumber nutrient solution (Ca(NO_3_)_2_ 1.75 mmol L^-1^, KNO_3_ 3 mmol L^-1^, NH_4_H_2_PO_4_ 0.5 mmol L^-1^, MgSO_4_⋅7H_2_O 1 mmol L^-1^). One repetition of each treatment consisted of two 1-L opaque plastic containers and each container contained four seedlings. Each treatment was repeated three times. The seedlings were grown in artificial climate chamber throughout the experiment. The light intensity in the climate chamber was 350–450 μmol m^-2^ s^-1^, temperature was 18–28°C and the relative humidity was 50–60%. The nutrient solution was changed at 2-day intervals.

### Experiments and Data Collection

In experiment 1, the 30-day-old seedlings which were raised in half-strength Yamasaki’s cucumber nutrient solution were used to determine the NaCl concentration that could cause moderate salt stress in the cucumber seedlings. The seedlings were subjected to five levels of NaCl concentrations including 0, 25, 50, 75, and 100 mmol L^-1^ for 10 days in the nutrient solution. The 50 mmol L^-1^ NaCl was found to have caused moderate salt stress in cucumber seedlings based on the morphological characteristics studied in experiment 1. In experiment 2, the most effective ALA (Sigma Aldrich, United States) concentration in alleviating moderate NaCl stress in cucumber seedlings was determined. There were five treatments including (1) normal nutrient solution only; (2) 50 mmol L^-1^ NaCl in nutrient solution + 0 mg L^-1^ ALA; (3) 50 mmol L^-1^ NaCl in nutrient solution + 10 mg L^-1^ ALA; (4) 50 mmol L^-1^ NaCl in nutrient solution + 25 mg L^-1^ ALA, and (5) 50 mmol L^-1^ NaCl in nutrient solution + 50 mg L^-1^ ALA. The ALA was applied with hand-held nebulize by thoroughly spraying both the upper and lower surfaces of leaves. ALA application was done twice, as soon as the seedlings were exposed to the salt treatment and at 24 h afterwards. Meanwhile, the treatments without ALA sprayed distilled water to the same extent. The 25 mg L^-1^ ALA was found to have the most appropriate alleviative effect towards moderate salt stress based on the morphological characteristics studied in the experiment 2. In experiment 3, after concentration screening of NaCl and ALA, the treatments were (1) CK: normal nutrient solution only; (2) NaCl: 50 mmol L^-1^ NaCl in nutrient solution + 0 mg L^-1^ ALA; (3) NaCl + ALA: 50 mmol L^-1^ NaCl in nutrient solution + 25 mg L^-1^ ALA, and (4) ALA: normal nutrient solution + 25 mg L^-1^ ALA. The ALA application was done as in experiment 2. All indexes were measured at 10 days after treatments application.

### Morphological Indexes

To selected appropriate concentration of chemicals (NaCl and ALA) in this study, leaf area and plant height of seedlings were measured at 10 days after treatment application. The leaf areas of fully opened true leaves of the seedlings were determined by a leaf area analyzer (YMJ-C, Tuopu Instruments Inc., China). Plant height was determined by tracing a string along the length of the stem and the length obtained was measured with a meter rule.

### Chlorophyll Fluorescence Parameters

Chlorophyll fluorescence induction parameters were measured using an Imaging-PAM Chlorophyll Fluorometer (Walz, Effeltrich, Germany). The seedlings from each treatment were kept in darkness for 30 min to fully open the reaction centers of photosystems II before measurement. Through the application of a saturation pulse, which was 2700 μmol m^-2^ s^-1^, Fo (minimum fluorescence of the dark-adapted leaves) and Fm (maximum fluorescence yield of the dark-adapted leaves) were obtained from dark-adapted leaves. Then, the actinic light (81 μmol m^-2^ s^-1^), which was opened every 20 s and lasted for 0.8 s, the light-adaptation time was 5 min. Under the application of actinic light, indexes like Fo’ (minimum fluorescence of the light-adapted leaves), Fs (steady chlorophyll fluorescence of light-adapted leaves) and Fm’ (maximum fluorescence yield of the light-adapted leaves) could be collected from the fully light adapted leaves.

The actual photosynthetic efficiency [Y(II)] was calculated as described by Genty et al. (1989). The quantum yield of regulated energy dissipation in PS II [Y(NPQ)] and the quantum yield of non-regulated energy dissipation in PS II [Y(NO)] were calculated according to Kramer et al.’s (2004) method. The coefficient of actinic light quenching (qL) was calculated according to the method of Klughammer and Schreiber (Klughammer and Schreiber, 2008). The specific computational formulas were as follows:

Y(II) = (Fm′-Fs)/Fm′

Y(NO) = 1/(NPQ+1+qL(Fm/Fo-1))

Y(NPQ) = 1-Y(II)-1/(NPQ+1+qL(Fm/Fo-1))

Y(II)+Y(NO)+Y(NPQ) = 1

NPQ = (Fm-Fm′)/Fm′

qL = (Fm′-Fs)/(Fm′-Fo′) × Fo′/Fs

### Gas Exchange Parameters

Gas exchange indexes including the net photosynthetic rate (Pn), intercellular CO_2_ concentration (Ci), stomata conductance (gs) and transpiration rate (Tr) of the fourth true leaf of sampled seedlings were determined with portable photosynthetic system (CIRAS-2, PP System, United Kingdom). The photosynthetic photon flux density (400 μmol m^-2^ s^-1^), ambient CO_2_ concentration (380 μmol mol^-1^), leaf temperature (25°C), and relative humidity (70%) were maintained throughout the measurements. In addition, the intrinsic water use efficiency (iWUE) of each treatment was derived based on Pn and gs according to Pimentel et al.’s (1999) method, which was calculated by dividing Pn by gs.

### Intermediates Contents on ALA Metabolic Pathway

#### ALA Content

The measurement of ALA was performed according to the methods of Morton (1975). A fresh leaf sample (5 g) was homogenized with 6 mL acetate buffer (pH 4.6) in ice bath; then, the homogenate was centrifuged at 5000 *g* for 15 min at 4°C. After that the supernatant (5 mL) was mixed with four drops of acetylacetic ester, and incubated at 100°C, 10 min for condensation reaction in a water bath. After cooling to room temperature, fresh Ehrlich’s reagent solution (containing 42 mL glacial acetic acid, 8 mL 70% perchloric acid, 1 g dimethylaminobenzaldehyde) in the same volume was mixed and allowed for 15 min. The absorbance was measured at 554 nm and concentration was calculated using a standard curve of ALA reference standards. Specifically, the concentrations of ALA standard curve were 0, 5, 15, 20, and 25 μg mL^-1^.

#### Uro III Content

Uroporphyrinogen III (Uro III) was determined according to Bogorad with some modifications (Bogorad, 1962). Fresh leaf sample (1 g) was homogenized with 5 mL Tris–HCl buffer (pH 7.2) in ice bath, and then the homogenate was centrifuged at 5000 *g* for 15 min at 4°C. The glacial acetic acid was used to adjust the supernatant to pH 4.0, then, centrifuged at 5000 *g* for 15 min at 4°C. The sediment was mixed with 5 mL distilled water and centrifuged at 5000 *g* for 15 min at 4°C. Then, precooled ammonia spirit (4 mL) was added into the sediment to extract Uro III, and centrifuged at 5000 *g* for 15 min at 4°C. The supernatant was evaporated to dryness at 55°C. Then, sulfuric acid-methanol (4 mL, 5%) was added into it to esterify for 48 h. Then, 20 mL distilled water was added to the mixture and extracted by mixing with 4 mL chloroform. The mixture was evaporated to dryness at 55°C, and then, chloroform (4 mL) was added and the absorbance was measured at 405 nm and the content of Uro III was calculated by the following formula. In the formula, 𝜀 = 5.48 × 10^5^ L mol^-1^ cm^-1^ is the molar extinction coefficient of Uro III under 405 nm. *d* = 1 cm is the optical path length of determine solution. *V* = 0.004 L is the dissolved volume of Uro III. FW = 1 g is the weight of fresh sample. The 10^9^ is to convert the unit from mol g FW^-1^ to nmol g FW^-1^.

Uro III (nmol g FW^-1^) = [A405/(𝜀 × d)] × V/FW × 10^9^

#### Proto IX, Mg-Proto IX and Pchlide Contents

The content of protoporphyrin IX (Proto IX), Mg-protoporphyrin IX (Mg-Proto IX), and protochlorophyllide (Pchlide) were determined according to the method of Hodgins and Van Huystee (1986) with some modifications. Fresh leaf sample (0.3 g) was homogenized with 5 mL 80% alkaline acetone, and then 80% alkaline acetone was added to the volume of 25 mL. The homogenate was incubated in dark condition until the tissue was bleached. After that, the homogenate was centrifuged at 1500 *g* for 10 min. The absorbance was measured at 575 nm, 590 nm, and 628 nm using the supernatant; then, the results were calculated by the corresponding formulas (Liu et al., 2015). In the formula, *V* is the dissolved volume of determined solution; *FW* is the weight of fresh sample.

$$\begin{array}{c} \mathrm{ProtoIX}(\mu mol g\;{\mathrm{FW}}^{-}{}^{1})\;=(0.18016\;\times \;A575-0.04036\;\times \;A628-0.04515\times \;A590)\times \;V/FW \end{array}$$

$$\begin{array}{c} Mg-ProtoIX(\mu mol\;g\;{\mathrm{FW}}^{-}{}^{1})\;=(0.06077\;\times \;A590-0.01937\;\times \;A575-0.003423\times \;A628)\;\times \;V/FW \end{array}$$

$$\begin{array}{c} \mathrm{Pchlide}(\mu mol\;g\;{\mathrm{FW}}^{-}{}^{1})\;=(0.03563\;\times \;A628+0.007225\;\times \;A590-0.02955\times \;A575)\;\times \;V/FW \end{array}$$

#### Heme Content

The measurement of heme was performed according to the methods of Marsh et al. (1963) with some modifications. The fresh leaf sample (2 g) was ground in liquid nitrogen, and then mixed with 5 mL of extract I (containing 0.5 mL 0.1 mol L^-1^ ammonia and 4.5 mL pure acetone). The mixture was centrifuged at 8000 *g* for 10 min. This process was repeated until the chlorophyll was completely removed. After that, extract II (5 mL; containing 80% acetone, 16% dimethyl sulfoxide and 4% concentrated sulfuric acid) was added into the sediment, and centrifuged at 8000 *g* for 10 min. The supernatant was mixed with 0.7 mL ethanol and the absorbance was determined at 386 nm. The heme concentration was calculated using a standard curve of heme reference standards. And the concentrations of heme standard curve were 0, 1, 3, 5, 7, and 10 μg mL^-1^.

#### Chlorophyll Content

The chlorophyll content of leaves was extracted with an 80% buffered aqueous acetone according to Porra et al.’s (1989) method. The supernatant was determined at 646 nm and 663 nm, and the content of chlorophyll (Chl *a* and Chl *b*) was calculated using the following formulas according to Lichtenthaler and Wellburn (1983).

Chl *a* (mg g FW^-1^) = (12.21 × A663-2.81 × A646) × V/FW

Chl *b* (mg g FW^-1^) = (20.13 × A646-5.03 × A663) × V/FW

### Total RNA Extraction and Gene Expression Analysis

Total RNA was extracted using TaKaRa MiniBEST Plant RNA Extraction Kit (TaKaRa Biomedicals, Japan) according to the manufacturer’s protocol. Synthesis of cDNA was executed with RevertAid First Strand cDNA Synthesis Kit (Thermo Scientific, United States). RNA solution (contain 2 μg RNA) and 1 μL oligo(dT)18 were added into PCR tube, and incubated under 65°C for 5 min in a thermal cycler (Bio-Rad, United States), and then rapidly cooled in ice. The PCR tube was then incubated under 42°C for 60 min in the thermal cycler with 4 μL 5× buffer, 2 μL 10 mmol L^-1^ dNTPs, 1 μL RNA inhibitor, and 1 μL reverse transcriptase. After that, the reverse transcriptase was inactivated under 80°C. The real time quantitative RT-PCR was implemented to analyze the expression of enzyme genes among ALA metabolic pathway in cucumber seedlings through a SYBR Premix Ex Taq II (Tli RNaseH Plus; TaKaRa Biomedicals, Japan). The cucumber *α-tubulin* gene was used as an internal control. Gene bank accession numbers of the sequences used to design the primers are shown in **Table 1**. The q-PCR test was executed in a fluorescence ration PCR instrument (LightCycler^®^ 96 System, Roche, United Kingdom). The reaction system contained 10 μL 2 × Tli RNaseH Plus, 0.8 μL forward primer, 0.8 μL reverse primer, 2 μL cDNA, 6.4 μL RNase Free dH_2_O. Samples for RT-qPCR were obtained from three seedlings for each treatment (*n* = 3) in each qPCR test, and every sample on the sample plate contained three wells of target gene and three wells of negative control (by adding components of the reaction system except for templet cDNA, and the templet was replaced by RNase Free dH_2_O). The PCR conditions were: initial denaturation at 95°C for 30 s, then cycle steps (40 t) at 95°C for 5 s, 60°C for 30 s and the melt curve conditions were 95°C for 5 s, 60°C for 60 s and 95°C. The last step of cooling was 30 s at 50°C. Each qPCR manipulation was replicated three times. Quantification analysis was performed by the comparative CT method (Livak and Schmittgen, 2001). The CT value of α-tubulin was subtracted from that of the target gene to obtain a ΔCT value. The average CT value of the CK sample in this experiment was subtracted from the ΔCT value to obtain a ΔΔCT value. Then the relative expression level to the control for each sample was expressed as 2^-ΔΔCT^.

**Table 1:** Primer sequences and Genebank accession number of the *HEMA1, HEMH, CHLH, POR, CAO* and α*-tubulin* gene sequences.

Gene symbol	Accession number	Forward primer (5′-3′)	Reverse primer (5′-3′)
*HEMA1*	D50407	5′-TTTGTCTCAGCATCGTGGAG-3′	5′-ATGTTGTGTGGCATCGTTGT-3′
*HEMH*	AB037113	5′-TGGAGTGTTGTTGCTGAACC-3′	5′-TTGGAGGAAACGGAACAATC-3′
*CHLH*	JW942287	5′-TTCGGTTGTGTCGCTTACTG-3′	5′-ACCAAAGGCAAAGCAACAAT-3′
*POR*	D50085	5′-AATGATCGACGGTGGTGAGT-3′	5′-CAAATGTTATGCCGGTTTCC-3′
*CAO*	AB512416	5′-AGCAGATTCCCTTCATGCAC-3′	5′-AAATGTTTGCTCCGTTGACC-3′
α-*tubulin*	AJ715498	5′-ACGCTGTTGGTGGTGGTAC-3′	5′-GAGAGGGGTAAACAGTGAATC-3′

### Chloroplast Ultrastructure Observation

Small pieces (about 1 mm^2^) of fresh leaf samples were fixed in 3% glutaraldehyde in 0.1 M phosphate buffer (pH 7.4) for 24 h at 4°C. The leaf samples were then fixed in 1% H_2_OsO_4_ for 5 h. After that, dehydration was carried out using graded ethanol series (70%, 80%, 90%, and 100%); then, acetone-infiltrated and embedded in Epon812 epoxy resin. Ultrathin sections were cut on a microtome (Leica EM UC6 ultra-microtome, Japan), and then stained with uranyl acetate and lead citrate for 15 min. Ultrathin sections of cucumber leaf was examined and photographed with a transmission electron microscope (TEM, Joel JEM-1230, Japan).

### Statistical Analysis

All the experiments were performed with three replicates and results were expressed as mean ± SE. Analysis of variance was performed using SPSS 22.0 (SPSS Institute Inc., United States) and treatments means were compared using the Tukey’s test at a 0.05 level of probability. All figures were prepared with OriginPro 2017 (OriginLab Institute Inc., United States).

## Results

### Leaf Area and Plant Height

**FIGURE 1:**
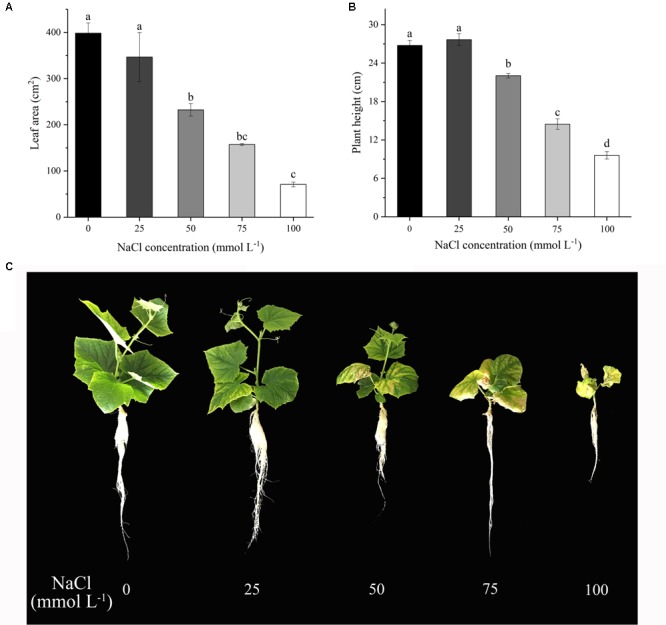
Effects of different NaCl concentrations on leaf morphology and plant height of cucumber seedlings. **(A)** Leaf area changes of cucumber seedlings under different concentration of NaCl. **(B)** Plant height of cucumber seedlings under different concentration of NaCl. **(C)** Morphological changes of cucumber seedlings under various concentration of NaCl, including 0, 25, 50, 75, 100 mmol L^-1^. The data presented were the means of three plants ± SE of means (*n* = 3, with 6 plants per replication). Treatment means with different letters indicate significant differences according to Tukey’s test (*p* < 0.05).

Salinity stress, which was administered by various concentrations of NaCl in 1/2 Yamasaki cucumber nutrient solution reduced the leaf area of seedlings (**Figure 1**). Except the 25 mmol L^-1^ NaCl treatment, leaf area, and plant height were decreased significantly with the higher concentrations of NaCl, where the highest concentration of NaCl (100 mmol L^-1^) even caused the death of the seedlings. Compared with the control, the 25-mmol L^-1^ NaCl caused 13.00% reduction in leaf area but increased plant height by 3.36%. The 50-mmol L^-1^ NaCl resulted in 41.67% reduction in leaf area and 17.68% in plant height. The 75-mmol L^-1^ NaCl decreased leaf area by 60.52% and plant height by 45.95%. The 100-mmol L^-1^ NaCl caused 82.21% reduction in leaf area and 64.13% in plant height. Based on the effects of the various NaCl concentrations on leaf area and plant height, we considered the 25 mmol L^-1^ as mild stress, 50 mmol L^-1^ as moderate stress, 75 mmol L^-1^ as severe stress and 100 mmol L^-1^ as lethal dosage. The 50-mmol L^-1^ NaCl treatment which provided moderate salt stress was employed in the subsequent experiment.

**FIGURE 2:**
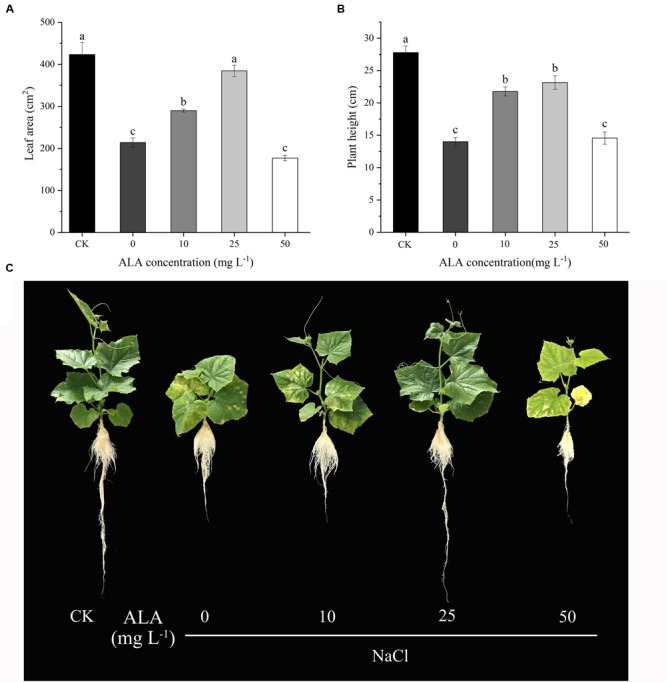
Effects of different ALA concentrations on leaf morphology and plant height of cucumber seedlings under salt stress. **(A)** Leaf area changes of cucumber seedlings treated with different concentration of ALA. **(B)** Plant height of cucumber seedlings treated with different concentration of ALA. **(C)** Morphological changes of cucumber seedlings under various ALA concentrations. The data presented were the means of three plants ± SE of means (*n* = 3, with 6 plants per replication). Treatment means with different letters indicate significant differences according to Tukey’s test (*p* < 0.05).

As shown in **Figure 2**, the effects of various concentrations of ALA sprayed on the seedlings showed dose-dependent effect under moderate salt stress. With the increase of ALA concentration, the leaf area and plant height of cucumber seedlings showed a tendency of increasing first and then decreasing. Moreover, both the two indexes reached the highest values when treated with 25 mg L^-1^ ALA under NaCl stress. Otherwise, the leaf area in 25 mg L^-1^ ALA treatment had no significant difference with that in CK treatment. However, cucumber leaves treated with high level of ALA (50 mg L^-1^) showed growth inhibition and chlorotic symptoms. Consequently, 25 mg L^-1^ ALA was the optimal concentration against moderate NaCl stress and was used for further experiments.

### Chlorophyll Fluorescence Parameters

**FIGURE 3:**
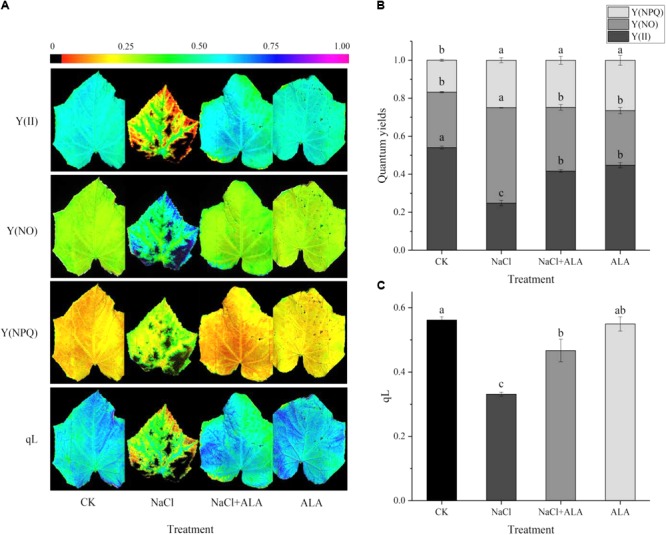
Effects of ALA on chlorophyll fluorescence parameters under salt condition. **(A)** Chlorophyll fluorescence images, the fluorescence images of the Y(II), Y(NO), and Y(NPQ) are given in corresponding colors that represent the absolute values of the ratio ranged from 0 (black) to 1.0 (purple). **(B)** Quantum yield of PSII, including Y(II) (actual photosynthetic efficiency of PS II), Y(NO) (the quantum yield of non-regulated energy dissipation) and Y(NPQ) (the effective quantum yield of PS II). **(C)** Changes of qL (photochemical quenching coefficient). The data presented were the means of three plants ± SE of means (*n* = 3). Treatment means with different letters indicate significant differences according to Tukey’s test (*p* < 0.05).

As shown in **Figure 3**, Y(II) and qL markedly declined in leaves treated with NaCl alone and Y(NO) increased significantly compared with CK. When the leaves were sprayed with ALA under salt condition, it significantly promoted Y(II) and qL of the seedlings but Y(NO) was suppressed to the level of CK. Furthermore, the Y(NPQ) of leaves in CK treatment was lower than those of the other treatments.

### Gas Exchange Parameters

**FIGURE 4:**
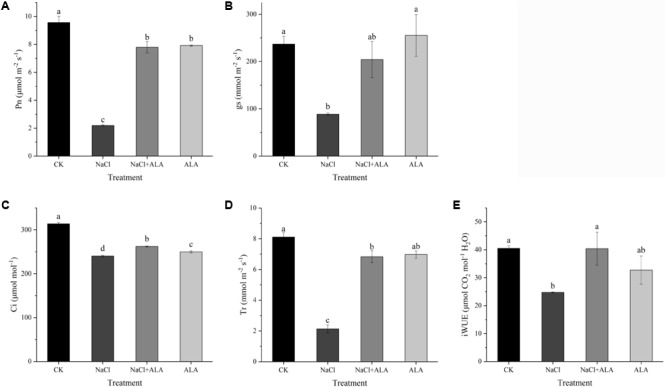
Effects of ALA on photosynthetic gas exchange parameters under salt stress. **(A)** Net photosynthetic rate (Pn). **(B)** Stomatal conductance (gs). **(C)** Intercellular CO_2_ concentration (Ci). **(D)** Transpiration rate (Tr). **(E)** Intrinsic water use efficiency (iWUE). The data presented were the means of 3 plants ± SE of means (*n* = 3). Treatment means with different letters indicate significant differences according to Tukey’s test (*p* < 0.05).

**Figure 4** showed the results on Pn, gs, Ci, Tr and iWUE of cucumber leaves under salt stress with or without ALA application. All the indexes were decreased by 50 mmol L^-1^ NaCl when compared to CK. Compared with seedlings treated with NaCl alone, application of ALA under salt condition showed positive effect on the photosynthetic gas exchange parameters, among them, Pn was increased by 255.84%, gs 130.50%, Ci 8.97%, and Tr 220.35%. Meanwhile, exogenous application of ALA stimulated the leave iWUE of seedlings which was depressed under salt stress. The value of iWUE increased by 63.28% compared to NaCl treatment. For the treatment with ALA alone, Pn and Ci decreased but gs and Tr showed no significant difference compared to CK.

### Intermediates Contents on ALA Metabolic Pathway

**FIGURE 5:**
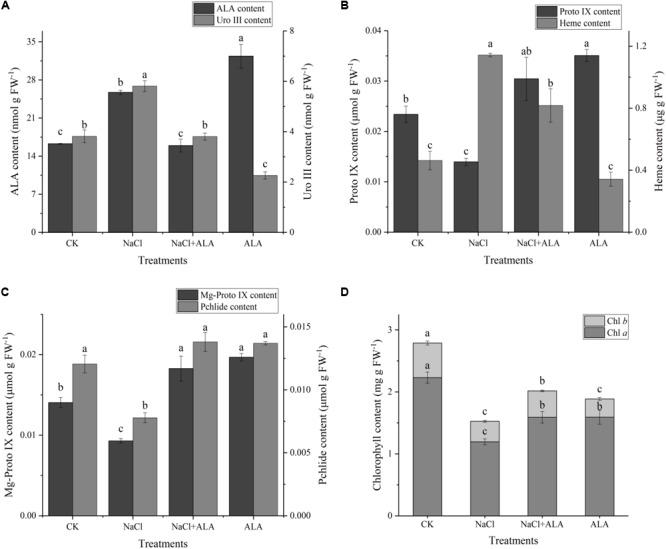
Contents of intermediates on ALA metabolic pathway. **(A)** Contents of ALA (5-aminolevulinic acid) and Uro III (uroporphyrinogen III). **(B)** Contents of Proto IX (protoporphyrin IX) and heme. **(C)** Contents of Mg-Proto IX (Mg-protoporphyrin IX) and Pchlide (protochlorophyllide). **(D)** Content of chlorophyll, including Chl *a* and Chl *b*. The data presented were the means of 3 plants ± SE of means (*n* = 3). Treatment means with different letters indicate significant differences according to Tukey’s test (*p* < 0.05).

The contents of several important intermediates among the ALA downstream metabolic pathway were determined to explore the mechanism of exogenous ALA on photosynthesis of cucumber seedlings under salt stress. As shown in **Figure 5A**, the contents of ALA and Uro III increased markedly under stressful condition, while, foliar application of ALA recovered ALA and Uro III contents to levels similar to that in the CK. Under salt stress, the Proto IX content reduced by 40.37% in contrast to the control group, but was remarkably increased when ALA was applied to seedlings. In seedlings treated with ALA alone, the content of Proto IX reached the highest value among all treatments (**Figure 5B**). On the contrary, the heme content significantly increased by 147.01% compared to CK under salt stress. When ALA was applied to seedlings subjected to the stress condition, the heme content decreased. Moreover, heme content showed no significant difference compared to CK when ALA was applied under normal growth condition (**Figure 5B**). The contents of Mg-Proto IXand Pchlide were both decreased by salinity but exogenous ALA application increased their levels evidently in cucumber leaves under salt stress. In addition, treatment with ALA only showed relative high concentrations of Mg-Proto IX and Pchlide (**Figure 5C**). Salt stress inhibited the total content of chlorophyll markedly, which was due to the reduction of both chlorophyll *a* and chlorophyll *b*. Exogenous application of ALA reversed the inhibitory effects of salinity on chlorophyll content. Besides, as for the treatment with ALA only, the contents of Chl *a* and Chl *b* were decreased compared to CK (**Figure 5D**).

### Relative Expressions of Genes on ALA Metabolic Pathway

**FIGURE 6:**
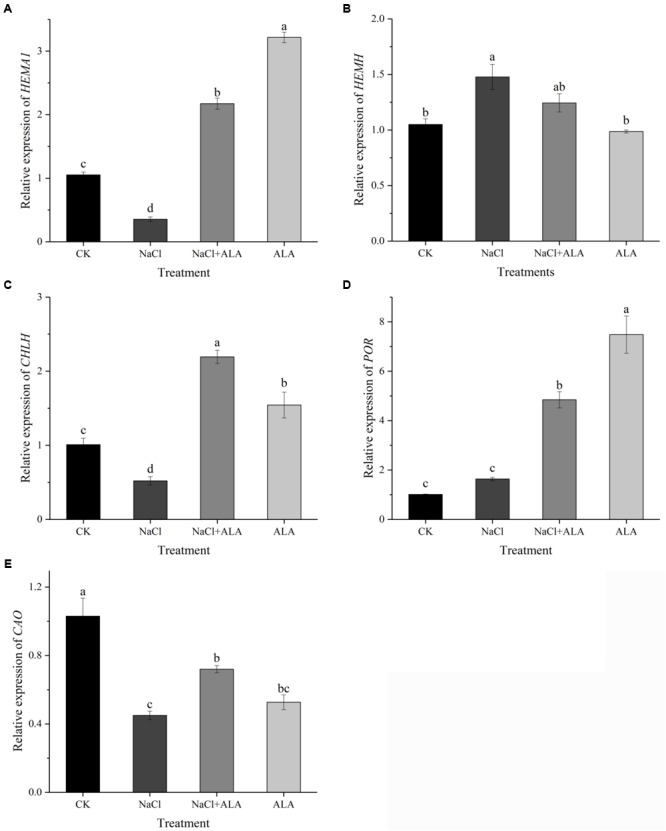
Relative expressions of genes involved in ALA metabolic pathway. **(A)** Relative expression of *HEMA1*, encoding glutamyl-tRNA reductase (Glu-TR). **(B)** Relative expression of *HEMH*, encoding ferrochelatase (FECH). **(C)** Relative expression of *CHLH*, encoding Mg-chelatase (MCH). **(D)** Relative expression of *POR*, encoding protochlorophyllide oxidoreductase (POR). **(E)** Relative expression of *CAO*, encoding chlorophyllide *a* oxygenase (CAO). The data presented were the means of 3 plants ± SE of means (*n* = 3). Treatment means with different letters indicate significant differences according to Tukey’s test (*p* < 0.05).

The relative expression of genes related to tetrapyrrol biosynthesis pathway is presented in **Figure 6**. Under salinity, the level of *HEMA1* was down-regulated significantly compared to untreated plants, but up-regulated by applying ALA under stress condition. In addition, treatment with ALA alone resulted in a threefold expression of *HEMA1* compared to control (**Figure 6A**). *HEMH* expression was up-regulated significantly by salt stress, and then showed slight decrease by spraying ALA. But *HEMH* gene level in seedlings treated with ALA solely had no significant difference compared to that CK (**Figure 6B**). Although suppressed by salinity, the expression of *CHLH* was stimulated by ALA application, which was 2.17-fold compared to control (**Figure 6C**). As shown in **Figure 6D**, the gene level of *POR* remained stable under moderate salt stress, while ALA treatments up-regulated its expression to 4.79 and 7.41 fold with or without stress condition, respectively, compared to the control. The expression of *CAO* was inhibited by salt but recovered by exogenous ALA. Moreover, *CAO* gene in treatment with ALA alone revealed relative low level compared with CK (**Figure 6E**).

### Ultrastructure Morphometric Changes

**FIGURE 7:**
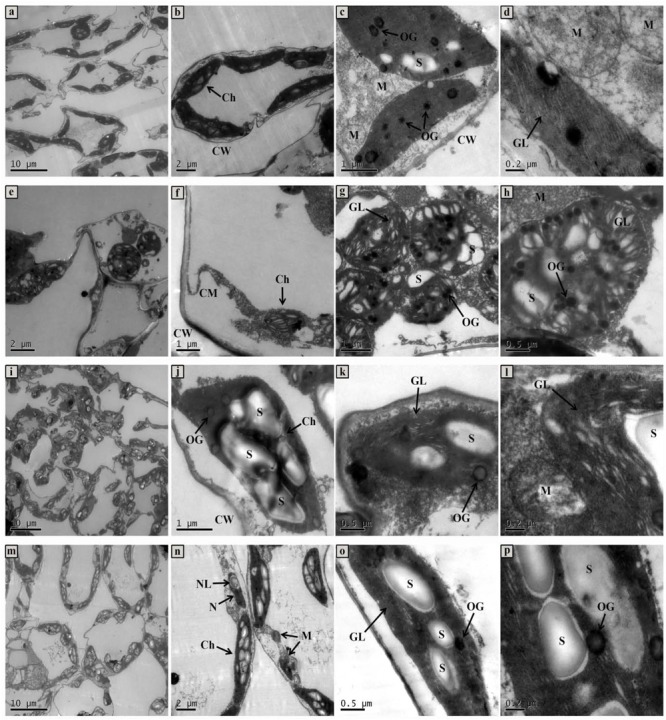
The ultrastructural observation of mesophyll cell and chloroplast of cucumber seedlings. **(a–d)** Seedlings grown in normal condition. **(e–h)** 50 mmol L^-1^ NaCl treated seedlings. **(i–l)** Seedlings treated with 50 mmol L^-1^ NaCl and 25 mg L^-1^ ALA simultaneously. **(m–p)** Seedlings sprayed 25 mg L^-1^ ALA only. Abbreviations: CW, cell wall; CM, cell membrane; N, nucleus; NL, nucleolus; Ch, chloroplast; OG, osmiophilic globules; GL, grana lamella; S, starch; M, mitochondria.

Changes of whole mesophyll cells and chloroplasts are shown in **Figure 7**. Seedlings grown in normal condition exhibited regular cell shape and typical chloroplast; meanwhile, there were smoothly arrayed grana lamellae, number of well packed starch grains, and a small quantity of osmiophilic granules (**Figures 7A–D**). However, cell morphological disturbance and plasmolysis occurred when seedlings were treated with 50 mmol L^-1^ NaCl, but the number of mitochondria markedly increased (**Figures 7E,F**). The grana lamellae of thylakoid were loosed and the shapes of chloroplasts severely swollen. Furthermore, there were plenty of osmiophilic granules as well as less starch grains in chloroplast compared with control (**Figures 7G,H**). For the ALA treated seedlings under salt stress condition, although there was a little improvement of cell morphology, the shapes of chloroplast become typically fusiform (**Figures 7I,J**). Moreover, the chloroplasts contained more orderly grana lamellae, more starch grains and fewer osmiophilic granules than those of seedlings under NaCl stress (**Figures 7K,L**). Under normal growth condition, ALA-treated seedlings were very similar to that of the CK plants. However, exogenous ALA application significantly increased the number of starch grains in chloroplast of the treated seedlings (**Figures 7M–P**).

## Discussions

Salt stress is one of the common abiotic stresses in agricultural production, and salinization in soil or culture substrates is a growing problem for agriculture worldwide (Deinlein et al., 2014). The harmful effects caused by rhizospheric salt stress involve various physiological and biochemical mechanisms related to plant growth and development. Accordingly, improving the growth and tolerance of plants under salt conditions is gaining prominence in research field.

### Alleviation Effects of Exogenous ALA on Growth Under NaCl Stress

In recent years, it has been well established that ALA, a natural plant growth regulator, could effectively improve plants tolerance to many environmental stresses (Akram and Ashraf, 2013). In the present study, we found that leaf growth of cucumber seedlings was suppressed by different NaCl concentrations of salt, and the inhibitory effect aggravated with the increase in salt level. However, application of ALA at a relative low concentration reversed the adverse effects caused by moderate NaCl stress. It has been reported that exogenous ALA increased plant dry weight, relative growth rate (RGR) and relative water content (RWC) under stressful conditions (Liu et al., 2014; An et al., 2016). In addition, ALA at 2 mg L^-1^ increased the hypocotyl length of oilseed rape under cadmium stress, but when ALA was added at 10 mg L^-1^, hypocotyl length decreased significantly (Ali et al., 2013a). These findings have shown that low levels of ALA stimulated the growth of seedlings, while high levels inhibited growth under stress condition.

### Regulation of Chlorophyll and Heme Biosynthesis Pathway by Exogenous ALA Under NaCl Stress

**FIGURE 8:**
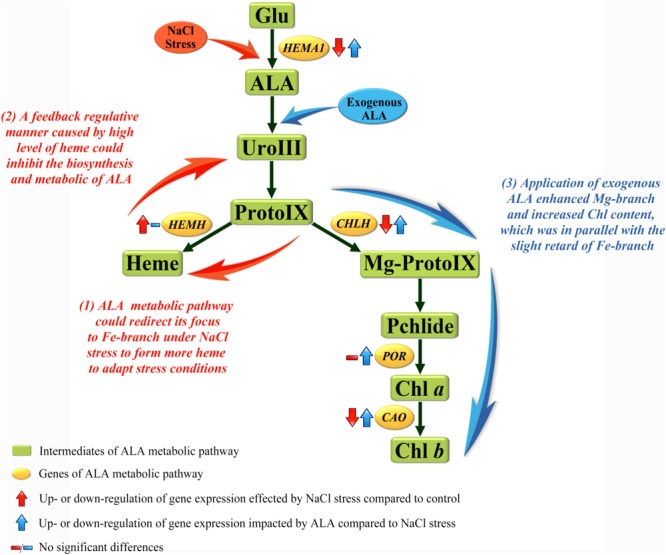
Regulation manners among ALA metabolic pathway of cucumber under NaCl stress. The metabolic pathway downstream of ALA in higher plants can mainly fall into Fe-branch and Mg-branch, for heme biosynthesis and chlorophyll biosynthesis, respectively. Results showed that (1) Fe-branch was enhanced under salt stress to form more heme, this could be conjectured as an adaptive or against response in plants; (2) The accumulation of heme under stress condition feed-back inhibited the biosynthesis and metabolic of endogenous ALA; (3) Application of exogenous ALA under stress enhanced the Mg-branch to produce more chlorophyll and kept the Fe-branch enhanced at the same time.

In order to explore the mechanism of the regulative effects of ALA on photosynthesis under stress, the pathway downstream of ALA, which is associated with photosynthetic pigment, was studied in the present research. The rate-limiting enzyme among this pathway which is encoded by *HEMA1*, Glu-tRNA reductase enzyme (GluTR), can be feedback regulated by downstream products. The relative expression of *HEMA1* was suppressed under salt condition, which was accompanied by increasing content of endogenous heme, indicating that the accumulation of heme restrained *HEMA1* gene expression by a feedback regulation. It has been demonstrated by other studies that reduction of *HEMA1* could weaken the activity of GluTR and inhibit heme and chlorophyll biosynthesis, while a notable quantity of heme could decrease the activity of GluTR (Kumar and Söll, 2000; Zhang M. et al., 2015). These results suggest that feedback inhibition manner of heme towards GluTR was mainly at the gene expression level. In addition, what made the large accumulation of heme under NaCl stress was the up-regulated *HEMH* gene which encoded ferrochelatase (FECH). The accumulation of heme was coupled with reduction of Proto IX, Mg-Proto IX and Pchlide, and we also noticed that endogenous ALA and Uro III accumulated at moderate salt concentration. It is possible to speculate that the metabolic pathway of ALA under moderate salt stress can redirect its focus from the pathway of chlorophyll branch to the heme branch; since the stress resistance effects of heme and its oxydates have been proven in higher plants. Catalyzed by heme oxygenase (HO), heme can be transformed into CO, free iron (Fe^2+^), and biliverdin (BV), then, BV will turn to bilirubin (BR) (Kwon et al., 2011). Among them, BR could inhibit protein oxidation *in vitro* in the presence of a variety of oxidants including superoxide and hydroxyl radicals; and CO played a critical role as signaling molecule and participated in regulating against various abiotic stress in plants (Wegiel et al., 2014; Wang and Liao, 2016). The study of transgenic rice (*Oryza sativa* L.), which overexpressed *Bradyrhizobium japonicum* FECH gene, resulted in increasing activity of FECH, raising content of heme and enhancing tolerance of oxidative stress (Kim et al., 2014). Apparently, this metabolic focus switch might be an adaptive mechanism in cucumber under NaCl stress (see regulation manner of metabolic pathway in **Figure 8**). In another study, water-deficit stress up-regulated the ferrochelatase gene and weakened the expression of Mg-chelatase gene in oilseed cotyledons (Liu et al., 2016b). Nevertheless, ALA applied exogenously reversed those phenomena caused by stress. For example, the expression of genes (including *HEMA1, CHLH* and *POR*) involved in chlorophyll biosynthesis was up-regulated under stressful condition. Especially for the gene *CHLH*, which encodes the key H-subunit of Mg-chelatase, is the crucial regulator in Fe-branch (Chen, 2014). MCH consists of three subunits, ChlH, ChlI, and ChlD in higher plants, among which it is ChlH that is primarily responsible for catalytic action of MCH (Richter and Grimm, 2013). The content of intermediate products in chlorophyll pathway, such as Proto IX, Mg-Proto IX, and Pchlide, were all increased by ALA application. Finally, chlorophyll *a* (Chl *a*) content was significantly increased by the strengthening of whole Mg-branch. Moreover, the content of chlorophyll *b* (Chl *b*) was also raised by up-regulating *CAO* gene which could be inhibited by salt. Chl *a* and Chl *b* are known as the major light-harvesting pigments in photosystems of all oxygenic photosynthetic organisms. Meanwhile, CAO is a unique enzyme responsible for Chl *b* synthesis (Sakuraba et al., 2010; Tsuchiya et al., 2012). Therefore, photosynthetic capacity of photosystems II (PS II) was enhanced by increasing light-harvesting pigments in our study. Exogenous ALA increased the content of chlorophyll in rice (*Oryza sativa* L.) (Nguyen et al., 2016). Subsequently, we also noticed that at stressful condition, heme content and *HEMH* expression were not reduced to the level of CK. Moreover, the Fe-branch of seedlings treated with ALA under normal condition remained stable similar to the level of control while the Mg-branch was stimulated markedly. This interesting phenomenon might suggest that stress environment is a prerequisite for heme pathway strengthening; and the effects of exogenous ALA in enhancing chlorophyll biosynthesis coordinated with the slight retard of heme biosynthesis (see regulation manner of metabolic pathway in **Figure 8**). Therefore, exogenous ALA can reverse the suppression of chlorophyll biosynthesis pathway which is caused by stressful conditions in plants. Meanwhile, the heme biosynthesis pathway can be enhanced under stress as an adaptive mechanism of the plant, and can also be retarded under enhanced chlorophyll synthesis.

### Promotion Effects of Exogenous ALA on Photosynthetic Capacity Under NaCl Stress

The effectiveness of photosystems II (PS II) can be reflected by chlorophyll fluorescence indexes. The present study has shown that saline rhizosphere and foliar application of ALA affected various chlorophyll fluorescence indexes of cucumber seedlings, including Y(II) (actual photosynthetic efficiency); Y(NO) (quantum yield of non-regulated energy dissipation in PSII) and Y(NPQ) (quantum yield of regulated energy dissipation in PS II) quantum yield of regulated energy dissipation in PS II) (Klughammer and Schreiber, 2008). Moreover, these three indexes represent the energy distribution and the activity of the photosynthetic reaction center in PS II. In this study, Y(II) and qL were significantly declined by NaCl stress while Y(NO) and Y(NPQ) were increased, suggesting that salt stress reduced the quantity of light quantum absorbed by the reaction center, and shut down PS II. The excess light energy couldn’t dissipate through regulatory mechanism of seedling, implicated by the high Y(NO), the damage had been caused to photosynthetical system under salt condition. These results were similar to the report on oil rape (*Brassica napus* L.), where herbicide stress aggravated the non-regulated heat dissipation and weakened photosynthetic efficiency (Jin et al., 2011). However, exogenous ALA effectively diminished the proportion of Y(NO) and enhanced photochemical energy conversion although Y(NPQ) remained stable. As mentioned above, under NaCl stress, application of ALA could enhance the relevant gene and intermediates of Mg-branch, which ultimately led to the increase in Chl *a* and Chl *b* contents. Therefore, excess light energy was reduced through the improved absorption and transmission of light energy since Chl *a* and Chl *b* were the main constituents in light-harvesting complex II (LHC II) (Akihito and Graham, 2010). Besides, photosynthetic efficiency in PS II of treatment with ALA alone was retarded, which was coupled with the reduction of chlorophyll content. This suggested that application of ALA to plants under normal growth environment would not enhance photosynthesis. In contrast, light-sensitive intermediate products (such as Proto IX and Pchlide) accumulated in thylakoid, and easily caused photo-damage (Lee et al., 2003). The reason behind the enhancement of light harvesting efficiency under salt stress is due to heightening of Mg-branch through applied-ALA which mainly increased the chlorophyll content.

Carbon assimilation is a vital procedure of photosynthesis in higher plants. Previous studies have shown that gas exchange indexes under salinity stress could be enhanced by ALA application (Liu et al., 2014). In our experiment, we found that ALA significantly increased the net photosynthetic rate and gas exchange capacity in cucumber seedlings under NaCl stress conditions. Salinity could inhibit the stomatal aperture and led to the reduction of gs. It has been demonstrated that under water-stress the aquaporins (such as *OePIP1.1* and *OePIP2.1*) are closely involved in the regulation of gs (Perez-Martin et al., 2014). Stomatal conductivity and net photosynthesis rate have been reported to be sensitive to saline environment (Nelson et al., 2007). Cadmium stress decreased the net photosynthetic rate by decreasing gs in oil seed rape but the ALA-treated plants exhibited distinct improvement of the gas exchange indexes and photosynthesis activity (Ali et al., 2013b). In another experiment, lead toxicity negatively affected Ci and Tr of *Brassica napus*, which were repaired by exogenous ALA (Tian et al., 2014). Relative high gs could increase CO_2_ uptake of mesophyll cell, and the assimilatory efficiency of Calvin cycle could be enhanced. Coupled with the photoreaction we discussed above, exogenous ALA enhanced photosynthetic efficiency in PS II under stress, which could bring more energy to dark reaction. Moreover, ALA reversed the reduction of gs and Ci caused by stress condition, which could bring more carbon source to form assimilation product. Therefore, net photosynthetic rate could be ameliorated by ALA under salt stress. The index of iWUE is often used to evaluate gas exchange adaptation. Researchers have found that it was closely related to environmental CO_2_ concentration for plants in nature (Leavitt et al., 2003; van der Sleen et al., 2014). In the facility environment with equal CO_2_ level, it could be regulated by stress conditions. For example, under mild water-deficit stress, the iWUE of *Phaseolus vulgaris* increased obviously, indicated that plants might have adaptation mechanism to against stress condition (Santos et al., 2009). However, under NaCl stress, in the present study, the value of iWUE in cucumber leaves decreased significantly. Similarly, in a pot-experiment that simulated salt stress in groundwater, with the increasing penetration depths of ground salinity water (0.3–0.8 m), the iWUE of quinoa (*Chenopodium quinoa* Willd.) continued to decrease (Talebnejad and Sepaskhah, 2016). Since cucumber seedlings were suffered in moderate salt stress for ten days, it indicated that the iWUE of plant is sensitive to stress degree. Nevertheless, exogenous ALA increased the iWUE of seedlings under stress condition. This indicated that ALA could enhance stomatal conductance and water use for photosynthesis then alleviated the harmful effects of salt stress. This promotive role of ALA might relative to the enhanced function of aquaporins (AQPs). Since the AQP genes could be up-regulated by exogenous ALA under salinity condition, and overexpression *NtAQP1* in tomato resulted in improved iWUE, because some aquaporin protein functions as both water and CO_2_ channel (Sade et al., 2010; Yan et al., 2014).

### Enhancement Effects of Exogenous ALA on Ultrastructure of Mesophyll Cell Under NaCl Stress

The alleviation effect of ALA on chloroplast under stressful condition has been well proved by researches (Naeem et al., 2012; Ali et al., 2013a; Tian et al., 2014). In this study, leaves of cucumber seedlings which were grown in normal condition had typical shape of mesophyll cell and chloroplast, whereas those under salt stress showed swollen chloroplast and abnormal cellular morphology. Under lead toxicity, the chloroplasts became swollen and ruptured; cell wall and cell membrane were diffused, but in the TEM micrographs of leaves treated with ALA, the adverse effects of lead were fixed (Tian et al., 2014). In addition, the accumulation of starch grains in chloroplasts indicated the activation of carbon assimilation of photosynthesis in plants (Wang et al., 2015). The number and size of osmiophilic globules can also be used as an indicator of thylakoid disintegration (Ding et al., 2015). Osmiophilic granules are regarded as the collection of lipid produced by thylakoid degradation. In the present study, osmiophilic granules accumulated obviously in chloroplast, suggesting that salt stress caused cell senescence to the mesophyll cells of cucumber. The exogenous ALA repaired the lamellar structure in chloroplast and gradually decreased the number of osmiophilic granules. This observation was consistent with an earlier report of a study conducted on *Brassica napus* L (Ali et al., 2013b). Besides, the treatment with ALA under normal condition, the starch grains were obviously accumulated more than ALA applied under stress. It could be suggested that the starch grains probably decomposed to form energy and osmotic adjustment substances which worked against abiotic stress. Therefore, application of ALA repaired the disturbance of thylakoid and the accelerated cell senescence of mesophyll cell caused by stressful condition, which provided a necessary site for the normal operation of photosynthesis in plant.

## Conclusion

The results of our experiments have demonstrated that the tetrapyrrol biosynthesis pathway downstream of ALA could redirect its focus to heme branch to adapt salt condition. In addition, the chlorophyll biosynthesis pathway was enhanced by exogenous ALA which was accompanied with the retarding of heme synthesis pathway under salinity stress. Exogenous ALA application enhanced the tolerance of the seedlings to salt stress through improvement in chlorophyll synthesis, light harvesting capacity, photosynthesis capacity, and also retarded thylakoid degradation. Therefore, application of ALA on cucumber seedlings could ameliorate the harmful effects caused by NaCl stress.

## Author Contributions

YW and JY conceived and designed the research. YW, XJ, and XZ conducted the experiments. YW, ZT, and TG analyzed the data and prepared the figures and illustrations. YW wrote the manuscript. WL, LH, and MD read the manuscript and made valuable inputs. All authors read and approved the submission of the manuscript.

## Conflict of Interest Statement

The authors declare that the research was conducted in the absence of any commercial or financial relationships that could be construed as a potential conflict of interest.
